# Association of biofilm formation with multi drug resistance in clinical isolates of *Pseudomonas aeruginosa*

**DOI:** 10.17179/excli2019-2049

**Published:** 2020-02-18

**Authors:** Nazia Abdulhaq, Zeeshan Nawaz, Muhammad Asif Zahoor, Abu Baker Siddique

**Affiliations:** 1 Department of Microbiology, Government College University Faisalabad

**Keywords:** multi drug resistant, biofilm formation, Pseudomonas aeruginosa, pslA gene

## Abstract

*Pseudomonas aeruginosa* is considered as foremost cause of hospital acquired infections due to its innate and plasmid mediated resistance to multiple antibiotics making it a multi drug resistant (MDR) pathogen. Biofilm formation is a pathogenic mechanism harbored by this pathogen which further elevates its resistance to antibiotics and host defense system. The aim of the present study was to evaluate the biofilm forming potential and distribution of *psl*A gene in multi drug resistant *Pseudomonas aeruginosa* isolates obtained from different clinical samples. A total of 200 different clinical samples were collected after obtaining written consent from the patients. The samples were subjected to isolation and identification of *P. aeruginosa* by standard microbiological procedures. Confirmation of isolates was done by polymerase chain reaction targeting *opr*L gene. Kirby Bauer method was performed for detection of MDR isolates. Congo red agar (CRA) test and Microtiter plate assay (MPA) for observing the biofilm forming ability and amplification of *psl*A gene was also performed on MDR isolates. The results showed that from 200 samples 52 (26 %) were *P. aeruginosa* and among them 20 (38.46 %) were MDR isolates. The CRA showed 23 (44.23 %) while MPA detected 49 (94.23 %) isolates as biofilm producers while all the MDR isolates showed biofilm formation by MPA method. The *psl*A gene was detected in all biofilm forming isolates while 90 % in MDR *P. aeruginosa*. It was concluded that biofilm forming *P. aeruginosa* are more resistant to tested antibiotics and biofilm formation is strongly associated with presence of *psl*A gene.

## Introduction

*Pseudomonas aeruginosa* is a threatening and emerging public health problem throughout the world particularly in developing countries. It is still a main cause of mortality and morbidity in humans (Hirsch and Tam, 2010[10]). It is ranked 4^th^ among the nosocomial pathogens and difficult to treat due to its resistant behavior against different antibiotic drugs (Khan et al., 2015[17]). *Pseudomonas aeruginosa *is a foremost cause of nosocomial infections, including urinary tract infections, pneumonia and bacteremia. It is also found in patients having burns, surgical, pus and accidental wounds (Khalid et al., 2017[15]). These infections become severe when the patients have impaired immune system (Pagani et al., 2004[23]).

Biofilms are structurally complex surface connected populations in which bacterial cells are enclosed by extra cellular polymeric substances (EPS) produced by their own self. These EPS are mostly exopolysaccharides, extracellular deoxyribonucleic acid and proteins (Ryder et al., 2007[28]). Biofilm formation contributes to pathogenesis of *P. aeruginosa *both in acute as well as chronic infection in clinical settings (Schaber et al., 2007[31]). The bacteria residing in biofilms are much more resistant to antimicrobial agents and host immune response compared to their planktonic counterparts, leading to prolonged or chronic infections which are difficult to eradicate (Mah et al., 2003[20]). Biofilm forming bacteria produce couple of extracellular polymeric matrices which attached the bacterial community together within the biofilm. The key components of biofilm matrix are polysaccharides because they contribute towards overall architecture of biofilm and make the bacterial population resistant to antibacterials (Wozniak et al., 2003[34]). There are about three exopolysaccharides reported to be involved in *P. aeruginosa* biofilm formation which includes alginate, Psl, and Pel (Ghafoor et al., 2011[7]). Among these exopolysaccharides, Psl is a mannose-rich polymer having an essential role in preliminary steps of biofilm formation (Ma et al., 2006[19]).

The biofilm production retards the antimicrobial therapy against bacteria because the biofilm develops a barrier which reduces the drug penetration leading to treatment failure as well as hindering the recognition of the microorganisms by immune system (Gil-Perotin et al., 2012[8]). In view of the above mentioned facts, the objective of the current study was to phenotypically and genotypically evaluate the biofilm production ability of multi drug resistant *Pseudomonas aeruginosa* from clinical samples.

## Materials and Methods

### Sample collection

A total (n=200) clinical samples were collected aseptically from different healthcare centers of Punjab, Pakistan consisting of burn wounds, surgical wounds, sputum, urine and blood samples which were shifted to laboratory as soon as possible. The samples were collected during January 2018 to December 2018 by obtaining the written consent from the patients after explaining them the purpose and objectives of the study.

### Ethical statement

This study was approved by Institutional Ethics Review Committee of Government College University Faisalabad, Pakistan under code GCUF/ERC/18/03B, and the samples were collected in accordance with international safety rules and ethical standards.

### Isolation and identification of Pseudomonas aeruginosa isolates

The samples were streaked on the plates containing Pseudomonas cetrimide agar (Oxoid, Uk) and incubated at 37 °C overnight aerobically. Isolates were identified on the basis of their colony morphology, cultural and biochemical characteristics (Ijaz et al., 2019[13]).

### Molecular confirmation of Pseudomonas aeruginosa isolates

All the identified isolates of *Pseudomonas aeruginosa* were confirmed by Polymerase chain reaction (PCR) using specific primer set targeting *opr*L gene *(opr*L-F 5'-ATGGAAATGCTG AAATTCGGC-3') and (*opr*L-R 5'-CTTCTTCAGCTCGACGCGACG-3') with the product size of 504 bp. First the DNA of all the bacterial isolates was extracted with the help of GeneJET Genomic DNA Purification Kit (Thermo Scientific, UK). The DNA was subjected to PCR using the conditions as described by Douraghi et al. (2014[4]). The PCR products were visualized in 1 % ethidium bromide stained agarose gel under (Slite 200W, Taiwan) Gel documentation system. 

### Antimicrobial susceptibility testing

The antibiotic susceptibility pattern of *P. aeruginosa *clinical isolates was evaluated by Kirby-Bauer disc diffusion method as recommended by the Clinical and Laboratory Standards Institute (CLSI) (Ilyas et al. 2016[14]) based on the zone of inhibition. The cultures were streaked on Mueller Hinton agar plates and incubated at 37 °C. The zone of inhibition was measured after cultures had been incubated overnight. The test was performed using standard antibiotic discs Ceftriaxone (30 μg), Gentamicin (10 μg), Ciprofloxacin (5 μg), Amikacin (30 μg), Amoxicillin clavulanic acid (30 μg) Ceftazidime (30 μg), Doripenem (10 μg), Meropenem (10 μg), Imipenem (10 μg) and Aztreonam (30 μg) (Oxoid).

### Phenotypic characterization of biofilm production

Biofilm formation was determined *in vitro *using Congo red agar and microtiter plate assay. The CRA preparation, inoculation and observation of results were done as described by Freeman et al. (1989[5]). Briefly, the inoculated plates having Congo red agar were incubated overnight aerobically at 37 °C to obtain isolated bacterial colonies. CRA-positive isolates appeared as black colonies, while CRA-negative strains remained red. 

In microtiter plate assay, overnight cultures were adjusted and diluted using tryptic soy broth (100 folds). Aliquots (250 μL) of each isolate suspension were then inoculated into a flat-bottom 96-well plate and incubated for 22-24 hours at 37 °C. Each well was washed twice with 250 μL of sterile saline followed by shaking to remove poorly adherent or non-adherent bacteria. The plates were then dried using heat which favors the fixation of biofilm. Each well was then stained with 250 μL of crystal violet (0.1 %) for 15-20 mins. After washing and drying of plates, the dye bound to the adherent cells in each well was dissolved with 250 μL of 50 % acetone. The optical density (OD) of the biofilms was measured at 594 nm with ELISA reader (BioRad, USA) as described by Stepanovic et al. (2000[32]). 

### Detection of the pslA gene

The PCR amplification of *psl*A gene was done with primers: (*psl*A-F, 5'-TGGGTCTTCAAGTTCCGCTC-3') and (*psl*A-R, 5'-ATGCTGGTCTTGCGGATGAA-3') generating an amplicon of 119 bp (Maita and Boonbumrung, 2014[21]). *P. aeruginosa *(PAO1) was used as positive control for the *pslA *gene. The PCR reaction mixture was prepared (25 μL) and following thermal conditions were applied. The preliminary denaturation was performed at 94 °C for 5 minutes. Then 35 cycles were performed (denaturation at 94 °C for 30 seconds, annealing at 52 °C for 40 seconds and extension at 72 *°*C for 50 seconds). The final extension was done for 10 minutes at 72 °C in a thermocycler device (BioRad, USA).

## Results

In the present study, a total of 200 clinical samples were collected from different hospitals of Punjab, Pakistan. The results showed that *Pseudomonas aeruginosa* was identified from (n=52) samples on the basis of biochemical tests and PCR method making the cumulative prevalence of 26 %. The distribution of *P. aeruginosa* isolates on the basis of type of samples was elaborated in Table 1(Tab. 1).

All the isolates of *P. aeruginosa* were subjected to antimicrobial susceptibility testing and highest resistance was found against Ceftriaxone (94.23 %), Meropenem (92.30 %), Imipenem (90.38 %) and Aztreonam (84.61 %) while the isolates were found most susceptible to Amikacin (38.46 %) and Ciprofloxacin (28.84 %). Among all the isolates, 20 (38.46 %) were found multi drug resistant (MDR) *Pseudomonas aeruginosa* as shown in Table 2(Tab. 2).

**Figure 1:**
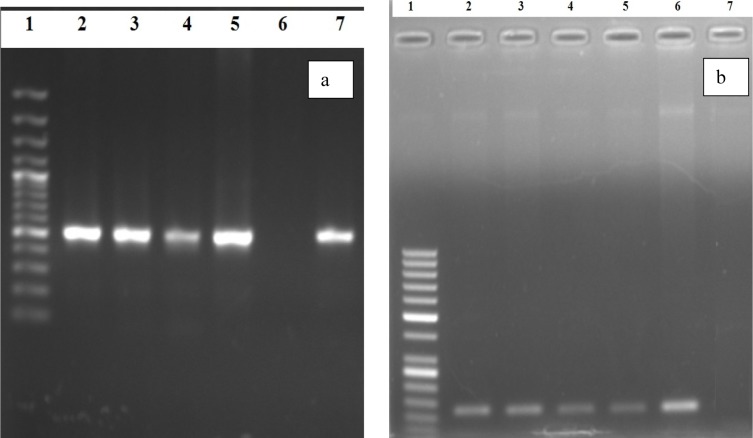
Polymerase chain reaction based detection of *Pseudomonas aeruginosa* *opr*L (a) and *psl* A (b) genes PCR based detection of (a) *oprL *(504 bp); Lane 1: 100 bp ladder, Lane 2-5: Positive Samples, Lane 6: Negative Control, Lane 7: Positive control for *oprL *genes confirming *Pseudomonas aeruginosa;* (b) *psl A *gene (119 bp); Lane 1: 50 bp ladder, Lane 2-5: Positive Samples, Lane 6: Positive Control, Lane 7: Negative Control for *psl A *gene

On the basis of Congo red agar test 23 (44.23 %) samples showed biofilm forming potential while when the Microtiter plate assay (MPA) was applied 49 (94.23 %) samples showed variable degree of biofilm production. Highest percentage was found (40.30 %) of moderate biofilm producing *Pseudomonas aeruginosa *followed by strong biofilm producers (36.50 %). All the (n=20) MDR *P. aeruginosa* isolates showed biofilm formation by MPA as shown in Table 3(Tab. 3). The *psl*A gene was detected in all biofilm forming isolates while (18/20) 90 % in MDR *Pseudomonas aeruginosa* isolates with polymerase chain reaction showing a 119 bp band on agarose gel as shown in Figure 1(Fig. 1).

## Discussion

*Pseudomonas aeruginosa *is an opportunistic pathogen having greatest concern for infections in various types of wounds, immunocompromised patients, and intensive-care units' patients (ICUs) (Bukholm et al., 2002[2])*. *Biofilm-producing *P. aeruginosa* have a key role in pathogenesis because biofilm decreased the penetration of antibiotics in bacteria and are difficult to remove from the surfaces. In the present study total 200 clinical samples were collected and the results showed 52 (26 %) found positive for *Pseudomonas aeruginosa* with most prominent 38.63 % in burn wound samples. The finding of our research was very close to the previous observations (20.05 %) by Samad et al. (2017[29]) in Peshawar and (22.2 %) by Hussain et al. (2017[12]) in Islamabad while our results were lower than the finding of Ijaz et al. (2019[13]) having 38 % prevalence of *P. aeruginosa*. Similarly the results of Khan et al. (2014[16]), Qureshi et al. (2018[26]) and Ijaz et al. (2019[13]) are consistent with our findings that *P. aeruginosa* was prominently found in burn wound/pus. The high prevalence of *Pseudomonas aeruginosa* is mainly due to elevated level of contamination, poor management of wounds and lack of sanitary facilities in hospital environment while the formation of pus in burn and surgical wounds serve as favorable condition for the growth of pathogens and transmission of infections (Procop et al., 2017[25]).

Multi drug resistant (MDR) refers to bacteria which show resistance to minimum two specific representatives of minimum two classes of antibiotics (Park et al., 2011[24]). The results of antimicrobial susceptibility pattern showed highest resistance of *P. aeruginosa* isolates towards Ceftriaxone (94.23 %), Meropenem (92.30 %), Imipenem (90.38 %) and Aztreonam (84.61 %) which are in accordance with the findings of Qureshi et al. (2018[26]) and Ijaz et al. (2019[13]) in Pakistan. Similarly Wang et al. (2010[33]) from China and Rodriguez-Martinez et al. (2009[27]) from France observed resistance towards beta lactams and carbapenems respectively. Among the positive *Pseudomonas aeruginosa* isolates 20 (38.46 %) were detected multi drug resistant (MDR). The results are lower than the observations of Maita and Boonbumrung (2014[21]) with 51 % and Ijaz et al. (2019[13]) with 58.6 % MDR strains of *P. aeruginosa*. The reasons behind such a resistance are the inappropriate or irrational use of antibiotics, mutation in the genome of *P. aeruginosa* and environmental conditions of the specific area.

In the present study, two different methods for detection of biofilm formation in *P. aeruginosa* were used and compared. It was observed that Congo red agar (CRA) test detected 23 (44.23 %) while the Microtiter plate assay (MPA) detected 49 (94.23 %) isolates as biofilm producers. The efficacy of MPA is better than CRA was also reported by Lima et al. (2017[18]) in Brazil and Samad et al. (2019[30]) in Pakistan. These results indicate that the biofilm formation carried out in liquid media gives more reliable results.

Among the total (n=52) *Pseudomonas aeruginosa* isolates, 49 (94.23 %) were detected to be positive for *psl*A gene and from the (n=49) biofilm forming *Pseudomonas aeruginosa,* all the isolates were found positive for *psl*A gene. Similarly among the 20 MDR *P. aeruginosa*, all were found positive for biofilm production by MPA but 18 (90 %) were found positive for *psl*A gene. Very similar results were previously obtained by Heydari and Eftekhar (2015[9]) and Abootaleb et al. (2020[1]) in Iran showing 100 % presence of *psl*A gene in biofilm forming *P. aeruginosa* while Ghadaksaz et al. (2014[6]) showed 83.7 % and Maita and Boonbumrung (2014[21]) showed 94 % prevalence of *psl*A gene. The presence of *psl*A gene had proven itself a good marker of biofilm formation in *Pseudomonas aeruginosa* isolates reported in previous findings by Overhage et al. (2005[22]), Ma et al. (2006[19]) and Hou et al. (2012[11]), but in some studies the *psl*A was also detected in non biofilm producing isolates (Heydari and Eftekhar, 2015[9]). It might be due to the fact that *psl*A plays an essential role in initial biofilm formation. So, in addition to *psl*A, there might be some other factors which play their role in biofilm formation (Colvin et al., 2011[3]).

## Conclusion

In conclusion, all the MDR isolates of *P. aeruginosa* showed biofilm forming potential and 90 % of them were positive for *psl*A gene. This presence of MDR strains along with biofilm formation is depicting a lethal combination of bacterial armory that poses a serious threat for public health.

## Acknowledgements

This study was partially funded by National Research Program for Universities (NRPU) of higher education Commission (HEC), Pakistan (5680/Punjab/NRPU/ R&D/HEC/2016).

## Disclosure

The authors declare that they have no conflict of interest. 

## Figures and Tables

**Table 1:**
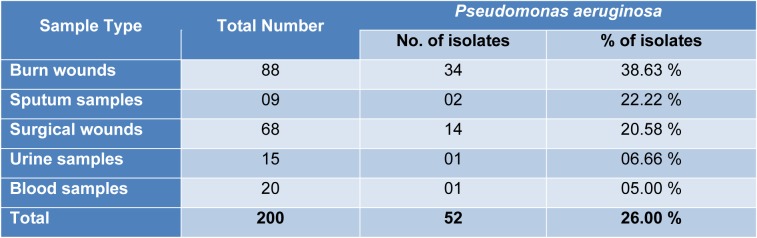
Distribution of *Pseudomonas aeruginosa* clinical isolates according to the sample type

**Table 2:**
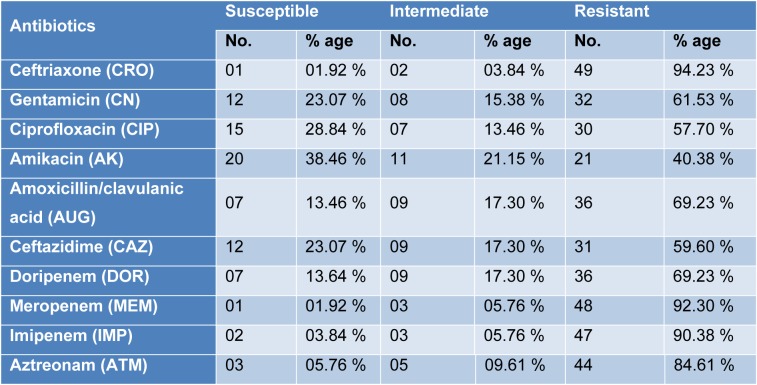
Antimicrobial susceptibility patterns of *Pseudomonas aeruginosa* isolates

**Table 3:**
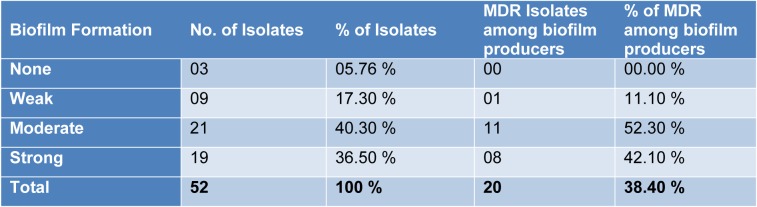
Association between biofilm forming and multi drug resistant isolates of *Pseudomonas aeruginosa*
